# Poverty, Income, and Unemployment as Determinants of Life Expectancy: Empirical Evidence from Panel Data of Thirteen Malaysian States

**Published:** 2020-02

**Authors:** Khaled TAFRAN, Makmor TUMIN, Ahmad Farid OSMAN

**Affiliations:** 1. Department of Administrative Studies and Politics, Faculty of Economics and Administration, University of Malaya, Kuala Lumpur, Malaysia; 2. Department of Applied Statistics, Faculty of Economics and Administration, University of Malaya, Kuala Lumpur, Malaysia

**Keywords:** Life expectancy, Poverty, Income, Unemployment, Income inequality, Healthcare commercialization

## Abstract

**Background:**

The primary indicator of public health, which all nations aim to prolong, is life expectancy at birth. Uncovering its socioeconomic determinants is key to extending life expectancy. This study examined the determinants of life expectancy in Malaysia.

**Methods:**

This observational study employs secondary data from various official sources of 12 states and one federal territory in Malaysia (2002–2014). Panel data of 78 observations (13 cross-sections at six points in time) were used in multivariate, fixed-effect, regressions to estimate the effects of socioeconomic variables on life expectancy at birth for male, female and both-gender.

**Results:**

Poverty and income significantly determine female, male, and total life expectancies. Unemployment significantly determines female and total life expectancies, but not male. Income inequality and public spending on health (as a percentage of total health spending) do not significantly determine life expectancy. The coefficients of the multivariate regressions suggest that a 1% reduction in poverty, 1% reduction in unemployment, and around USD 23.20 increase in household monthly income prolong total life expectancy at birth by 17.9, 72.0, and 16.3 d, respectively. The magnitudes of the effects of the socioeconomic variables on life expectancy vary somewhat by gender.

**Conclusion:**

Life expectancy in Malaysia is higher than the world average and higher than that in some developing countries in the region. However, it is far lower than the advanced world. Reducing poverty and unemployment and increasing income are three effective channels to enhance longevity.

## Introduction

**Fig. 1:**
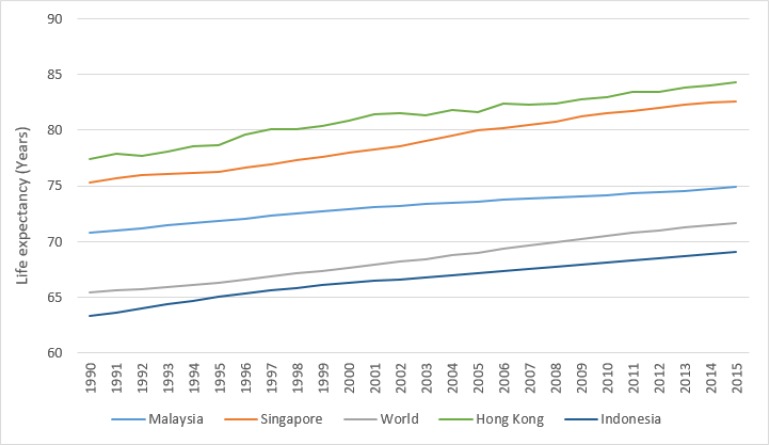
Trend of life expectancy in Malaysia compared to world average and some developing and developed Asian countries (Source: Word Development Indicators Database of the World Bank (1))

One of the most important indicators used as a measure of health status is life expectancy at birth. The highest worldwide life expectancy at birth is reported in Hong Kong (84.27 yr, in 2015; Fig. 1) (1). Life expectancy worldwide seems to be increasing rapidly, with recent research projecting that life expectancy at birth would reach 90 yr in South Korea by 2030 (2).

In Malaysia, life expectancy has improved significantly over the last few decades, from 70.

yr in 1990 to 74.87 yr in 2015 (Fig. 1). The latest available statistics (from 2015) show that Malaysia has higher life expectancy than the world average (71.66 yr), as well as higher life expectancy than some developing countries in the region, such as Indonesia (69.07 yr). However, Malaysian life expectancy still trails that of advanced countries, including Malaysia’s closest neighbor, Singapore (82.59 yr) (1). Comparison of Malaysia with other countries shows that although Malaysia has achieved relatively good life expectancy, it still has a long journey ahead to approach the health status of the advanced world (1).

Socioeconomic variables explain a large portion of variation in life expectancy (3–5). Factors such as poverty (3,4,6), income (7–12), unemployment (13–16), and income inequality (10,17) have all been found to predict variations in life expectancy in several studies of regions, nations, and economic clusters. However, the results of these studies have not always been consistent (17), suggesting that the socioeconomic determinants of life expectancy vary across regions, nations, and level of economic development. Thus, each country might have its own set of determinants of life expectancy (16).

In the Malaysian context, the commercialization of health care influences the health status of the Malaysian population, especially the poor (18). The share of public health spending as a percentage of total health spending dropped from about 90% before the late 1980s to a steady level around 55% in the late 1990s and thereafter (18,19). Moreover, the share of public health spending varies across Malaysian states, which have different levels of longevity. The commercialization of health care (or, conversely, the share of public health spending) is thus a policy variable expected to influence life expectancy in Malaysia.

Against this background, this study aimed to answer the following question: what determines life expectancy in Malaysia?

## Materials and Methods

### Data collection

For this observational study, the literature suggested the following variables of interest for the Malaysian scenario: life expectancy, poverty, income, income inequality, unemployment, and health care commercialization. Cross-sectional data on these variables were collected for the state level (for the 12 Malaysian states and one federal territory) at six points in time.

The outcome variables for this study are life expectancies at birth for female, male, and both genders. We measure poverty as the fraction of the population living under the national poverty line index, defined as “the minimum income needed by a household to meet the basic needs of food and non-food for each of its members to enable them to have a healthy and comfortable life.” (20). For 2014, the poverty line index was estimated at MYR 930 per household per month (20), or USD 216.30 with 2017 exchange rates.

Average household income in our sample represented income. To avoid biased estimates caused by time-variant price levels, we adjusted household income for inflation using a GDP (Gross Domestic Product) deflator. We obtained yearly deflator data from the World Development Indicators Database of the World Bank (1). As a proxy for income inequality, we used the GINI coefficient, which ranges from 0 (perfect equality) to 1 (perfect inequality). Our measure of unemployment was the proportion of the unemployed population to the total population in the labor force (aged 15–64 yr). The percentage of public health expenditure out of total health expenditure was the proxy for health commercialization, with a higher share of public spending on health care reflecting less commercialization.

We collected these data from various sources. Data on life expectancy came from the Malaysian Department of Statistics (21). Data on poverty, income, and income inequality came from the Malaysian Economic Planning Unit (22). Data on public health expenditures were compiled from the Malaysian National Health Accounts published by the Ministry of Health Malaysia and another source (23,24).

Malaysia has thirteen states and three federal territories. Data on life expectancy and the other independent variables were available for 12 states (Johor, Kedah, Kelantan, Melaka, Negeri Sembilan, Pahang, Perak, Perlis, Pulau Pinang, Selangor, Sarawak, and Terengganu) and for the federal territory of Kuala Lumpur, thirteen areas that account for 87.8% of the Malaysian population.

Data on life expectancy and unemployment were available between 2001 and 2015, but data on poverty, income, and income inequality were available only for specific years during which the Malaysian household national survey was conducted. We could match all data for six years: 2002, 2004, 2007, 2009, 2012, and 2014. Overall, then, we completed a panel of 78 observations (13 cross-sections at six points in time).

### Statistical analysis

Bivariate associations between variables were initially assessed using Pearson correlation tests. Next, we performed three multivariate regressions to examine the adjusted determinants of life expectancy for females, males, and both genders. Significance was set at the 5% level to accept or reject the null hypothesis. All regressions were cross-section, fixed-effect models since the Wald test supported fixed effects and the results of the Hausman test revealed no strong evidence for random effects. We found no evidence for using two-way, fixed-effect models because time dummies were not significant. We detected some heteroscedasticity, so for all regressions, we used standard errors and variance consistent with White heteroscedasticity. Notably, the 78 observations suffice for the above-described analyses, since the five independent variables with 12 dummies representing the state-based fixed-effects leave sufficient degrees of freedom for the residuals, which is good enough to obtain robust estimates. Some previous studies have used less than half this number of observations (16,25). Statistical analyses were performed using EViews 9 (IHS Global Inc., Irvine, CA, USA).

This study was approved by the University of Malaya Research Ethics Committee (Reference No: UM.TNC2/UMREC-164).

## Results

Table 1 presents descriptive statistics for the variables included in this study. The mean (± standard deviation) of both-gender life expectancy was 73.24 ±1.55 yr. Female average life expectancy (75.79±1.28 yr) was 5.1 yr higher than male life expectancy (70.69±1.88 yr; Table 1).

**Table 1:** Descriptive statistics of the study variables

***Variable***	***Mean***	***Median***	***Max***	***Min***	***Std. Dev.***
Life Expectancy at birth, Male, years.	70.69	70.50	74.60	67.20	1.88
Life Expectancy at birth, Female, years.	75.79	75.85	78.40	72.60	1.28
Life Expectancy at birth, Total, years.	73.24	73.18	76.45	69.95	1.55
Poverty (proportion of people under national poverty line), percentage.	3.22	1.60	17.80	0.00	3.79
Unemployment rate, percentage.	3.00	3.00	4.60	0.50	0.74
Monthly household Income,'000 MYR	3.77	3.42	10.37	1.62	1.63
Income inequality, GINI coefficient.	0.40	0.40	0.47	0.32	0.03
Public Health Expenditure (proportion, out of total health expenditure).	0.57	0.60	0.76	0.29	0.13

Max= Maximum, Min = Minimum, Std. Dev. =Standard deviation, MYR= Malaysian Ringgit (USD 1 ≈ MYR 4.3, as in Sep 2017)

The highest total life expectancy recorded was in Sarawak (73.24 yr) in 2014, and the lowest was in Terengganu (69.95 yr) in 2002. Average incidence of poverty in the sample was 3.22%, with the highest level in the state of Kelantan (17.80%) in 2002 and lowest in Johor (0.00%) in 2014. Unemployment ranged between 0.50% and 4.60%, with an average of 3.00%. Monthly household income ranged between MYR 10,367 (around USD 2,411), reported in Kuala Lumpur in 2014, and MYR 1,621 (around USD 376), observed in Kelantan in 2002, with an average in the sample of MYR 3,771 MYR (around USD 877). Average share of public expenditure on health (out of total health spending) was 56.64%, with the highest observation in Kelantan (76.11%) in 2012 and lowest in Selangor (28.90%) in 2004.

Bivariate analyses highly correlated total, male, and female life expectancies, with correlation coefficients all above 0.93. Life expectancies (total, female, and male) were significantly correlated with poverty, income, and share of public health expenditure (*P*<0.001). No life expectancy variables (total, female, and male) were correlated with income inequality (GINI; *P*>0.05). Only female life expectancy was significantly correlated with unemployment at the 0.05 significance level; meanwhile unemployment was correlated with neither male life expectancy nor with both-gender life expectancy. The coefficients of correlation across independent variables were all below 0.64, indicating no serious collinearity problems in our prospective multivariate models (Table 2).

**Table 2:** Bivariate correlation, Pearson correlation, across variables

	***LE (Female)***	***LE (Male)***	***Poverty***	***Income***	***GINI***	***Unemployment***	***Health Expenditure***
LE (Total)	0.977 (0.000)	0.989 (0.000)	−0.594 (0.000)	0.763 (0.000)	−0.064 (0.576)	−0.214 (0.060)	−0.509 (0.000)
LE (Female)	_	0.936 (0.000)	−0.658 (0.000)	0.771 (0.000)	−0.165 (0.149)	−0.267 (0.018)	−0.488 (0.000)
LE (Male)	_	_	−0.535 (0.000)	0.738 (0.000)	0.006 (0.960)	−0.172 (0.132)	−0.511 (0.000)
Poverty	_	_	_	−0.633 (0.000)	0.458 (0.000)	0.388 (0.000)	0.420 (0.000)
Income	_	_	_	_	−0.204 (0.074)	−0.343 (0.002)	−0.483 (0.000)
GINI	_	_	_	_	_	0.404 (0.000)	0.122 (0.288)
Unemployment	_	_	_	_	_	_	0.525 (0.000)

LE = Life Expectancy at birth.

*Note:* Values in parentheses are *P*-values

Table 3 presents the results of three multivariate regressions in which total, female, and male life expectancies are the dependent variables. Poverty significantly predicted total (β (coefficient) =−0.049, *P*=0.010), female (β= −0.056, *P*=0.003), and male (β= −0.044, *P*=0.035) life expectancies. Income also significantly predicted total (β=0.447, *P*<0.001), female (β=0.445, *P*<0.001), and male (β=0.465, *P*<0.001) life expectancies. Unemployment was a significant predictor only for total (β= −0.197, *P*=0.041) and female (β= −0.245, *P*=0.018) life expectancy, not for male (in earlier estimated model before being dropped and the model re-estimated).

**Table 3:** The influence of poverty, income, and unemployment on life expectancy in Malaysia; fixed effect multivariate regressions

***Variable***	***Life Expectancy (Total)***			***Life Expectancy (Male)***			***Life Expectancy (Female)***		
	***β***	***[SE]***	***(P-value)***	***β***	***[SE]***	***(*P*-value)***	***β***	***[SE]***	***(P-value)***
Poverty	−0.049	[0.018]	(0.010)	−0.044	[0.020]	(0.035)	−0.056	[0.018]	(0.003)
Income	0.447	[0.071]	(0.000)	0.465	[0.075]	(0.000)	0.445	[0.068]	(0.000)
Unemployment	−0.197	[0.094]	(0.041)	_	_	_	−0.245	[0.101]	(0.018)
Constant	74.760	[0.526]	(0.000)	72.126	[0.367]	(0.000)	76.768	[0.516]	(0.000)
Sarawak	Ref.	_	_	Ref.	_	_	Ref.	_	_
Johor	−2.173	[0.176]	(0.000)	−2.549	[0.150]	(0.000)	−1.664	[0.182]	(0.000)
Kedah	−2.531	[0.158]	(0.000)	−3.384	[0.138]	(0.000)	−1.589	[0.174]	(0.000)
Kelantan	−3.818	[0.169]	(0.000)	−5.009	[0.195]	(0.000)	−2.492	[0.174]	(0.000)
Melaka	−2.898	[0.301]	(0.000)	−3.224	[0.230]	(0.000)	−2.264	[0.317]	(0.000)
Negeri Sembilan	−2.823	[0.146]	(0.000)	−3.784	[0.195]	(0.000)	−1.807	[0.137]	(0.000)
Pahang	−2.706	[0.156]	(0.000)	−3.423	[0.158]	(0.000)	−1.849	[0.152]	(0.000)
Perak	−2.175	[0.269]	(0.000)	−3.186	[0.284]	(0.000)	−1.075	[0.253]	(0.000)
Perlis	−2.679	[0.128]	(0.000)	−3.587	[0.151]	(0.000)	−1.676	[0.175]	(0.000)
Pulau Pinang	−2.147	[0.250]	(0.000)	−2.564	[0.207]	(0.000)	−1.474	[0.248]	(0.000)
Selangor	−1.870	[0.223]	(0.000)	−1.928	[0.228]	(0.000)	−1.693	[0.231]	(0.000)
Terengganu	−4.242	[0.142]	(0.000)	−5.148	[0.174]	(0.000)	−3.240	[0.141]	(0.000)
F. Kuala Lumpur	−1.820	[0.408]	(0.000)	−1.840	[0.463]	(0.000)	−1.704	[0.363]	(0.000)
R^2^ (Adjusted-R^2^)		0.950	(0.938)		0.958	(0.948)		0.926	(0.908)
F-statistic (*P*-value)		78.232	(0.000)		101.962	(0.000)		51.932	(0.000)
No. of observations (No. of states)		78 (13)			78 (13)			78 (13)	
P-value of fixed-effect specification test		0.000			0.000			0.000	

SE = Standard error, F. = Federal territory, Ref. = Reference.

*Note:* All standard errors and covariance are White-heteroscedasticity-consistent

The states’ dummy variables showed that all states have lower total, female, and male life expectancies than the state of Sarawak, with all dummies significant at *P*<0.001. The lowest intercept was for the state of Terengganu and the highest was for the state of Sarawak.

The coefficients of the multivariate regressions suggest that a 1% decrease in poverty, MYR 100 increase in income and 1% decrease in unemployment prolong total life expectancy by 17.9, 16.3, and 72 d, respectively (Table 4). Moreover, the magnitudes of the effects of the socioeconomic variables on life expectancy vary some what by gender. Namely, poverty has stronger influence on life expectancy among women than men; a reduction in poverty by 1% prolongs life expectancy by 20.6 d for women but only 15.9 d for men. Household income has slightly larger effects on male life expectancy than females. An increase in household income by MYR 100 extends female life expectancy by 16.2 d, compared to 17.0 d for male life expectancy. Female life expectancy increases by 89.5 d in response to a 1% decrease in unemployment, while unemployment has no significant effect on male life expectancy.

**Table 4:** Magnitudes of the effects of socioeconomic variables on life expectancies, calculated based on the coefficients of the multivariate regressions^a^

***Change in variable***	***Improvement in life expectancy (days)***		
	***Total***	***Male***	***Female***
1% reduction in poverty	17.9	15.9	20.6
MYR 100 (≈ USD 23.2) increase in household income.^b^	16.3	17.0	16.2
1% reduction in unemployment	72.0	-	89.5

MYR= Malaysian Ringgit (USD 1 ≈ MYR 4.3, as in September 2017)

Notes:

a We calculated the magnitudes [M] based on the formula: M = D * β * 365; where D is the unit change in the independent variable, β is the respective coefficient of the independent variable (As presented in Table 3), and 365 is the number of days of a single year. We used days instead of years for clearer comparisons of the magnitudes across variables and genders.

b The initial unit of measurement for income in the regressions (Table 3) is thousand; however, for better illustration of the magnitudes, we used MYR 100 change in income in this table [D=0.1]

## Discussion

### Increasing income, extending longevity

**Fig. 2:**
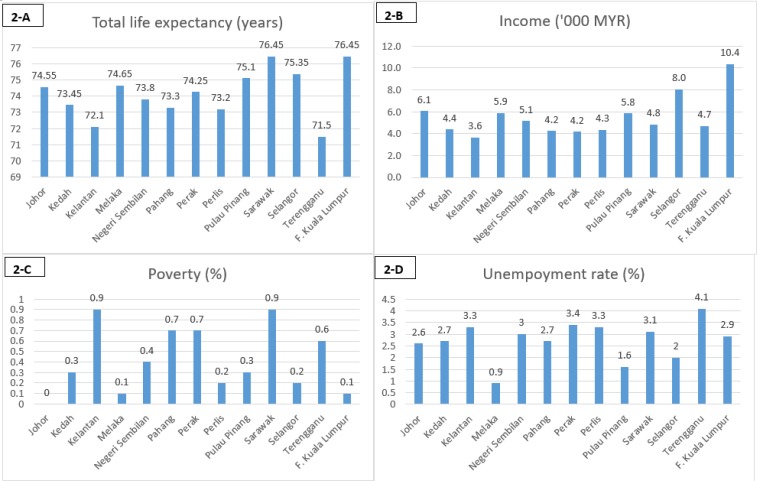
Latest statistics (from 2014) on life expectancy (A), income (B), poverty (C), and unemployment (D); by state. F. =Federal territory, MYR =Malaysian Ringgit (1 $USD ≈ 4.3 MYR, as in Sep 2017) Sources: Department of Statistics Malaysia (21) and the Economic Planning Unit Malaysia (22)

According to the results of this study, an increase of MRY 2200 (around USD 512) in monthly household income would add approximately one extra year to overall life expectancy across the country. Improving income is an effective method to prolong life expectancy in Malaysia, especially in states with relatively low household incomes and accordingly lower life expectancies (Fig. 2 A–B).

The results accord with most of the literature found positive effects of income on life expectancy (7–12). In fact, income affects health—and thus life expectancy—via various channels. Higher-income reflects better social status, strongly linked to better physical and mental health (10, 17, 26, 27). Much evidence documents that increasing income also improves access to health care (28, 29). People with higher income adopt more-healthy lifestyles (30, 31).

### Towards “zero” poverty and a healthier nation

Poverty is the root of many diseases and causes of mortality; reductions in poverty levels have been widely linked to better health outcomes (25, 32, 33). Moreover, and in line with the results of this study, poverty has a significant, negative effect on life expectancy (3,4,6).

Over the last decade, the prevalence of poverty in Malaysia was reduced from 6% in 2002 to 0.6% in 2014. The results of this study suggest that poverty eradication associates with better public health outcomes, higher life expectancy. The latest available statistics (2014) showed that the incidence of poverty reached zero in one Malaysian state (Johor; Fig. 2-C). However, poverty remains a minor problem in other states. The highest incidence of poverty (in 2014) was reported in the states of Kelantan and Terengganu (0.9%). Therefore, life expectancy could be improved by achieving “zero” poverty in all states; according to our results, eliminating the remaining 0.6% of Malaysian poverty would prolong life expectancy by about 10.7 d.

### More employed women, longer life expectancies

Unemployment evidently worsens health status, thus increasing mortality and undermining life expectancy (13–16). Moreover, the negative effect of unemployment is stronger on women’s health than men’s (15). As for life expectancy in Malaysia, our results confirm that unemployment negatively affects female life expectancy, but not male.

At a national level, Malaysian unemployment has held nearly steady around 3% over the last decade (1). Although this level of unemployment is deemed healthy from an economic perspective, it would not seem to optimize national life expectancy. A further reduction in the unemployment rate from 3% to about 0.9% (as in the state of Melaka in 2014, Fig. 2-D) would extend female life expectancy by about half a year and total life expectancy by approximately five months. However, the economic side effects (increased inflation) of such a reduction in unemployment should also be considered (34).

### The hidden effect of inequality

The effect of inequality on health remains controversial in the literature. Inequality had negative effects on life expectancy (10,17). Oher studies, however, have found no evidence for such an effect (35–37). The insignificant effects of inequality on health found by several studies could be attributed to indirect effects of inequality on health through other variables (mediators) (17). For instance, inequality increases poverty (38), and poverty leads to lower life expectancy; inequality thus may reduce life expectancy by increasing poverty. Moreover, inequality is well established to undermine income (39); inequality thus indirectly reduces life expectancy by reducing income.

### Which sector spends on health care is irrelevant

Public sector health spending comprised more than 90% of overall Malaysian health spending until the late 1980s. Later, a new policy was implemented that aimed to partially commercialize the health care system, decreasing public share of health spending to about 55% in the late 1990s and thereafter (18,19).

Our multivariate analyses revealed that the contribution of the public sector to health spending has no significant effects on life expectancy. Alternatively, the level of commercialization in the health sector has no effects on life expectancy.

### Limitations

Due to limited data availability, about 12.2% of the Malaysian population were not included in this study. Regression estimates might have varied slightly if missing data were available and included. However, the results of this study remain valid and generalizable, since the employed sample represents a very large fraction (87.8%) of the Malaysian population.

## Conclusion

Malaysia has achieved relatively high health status, reflected by a life expectancy that exceeds the world average and some countries in the region. Nonetheless, life expectancy in Malaysia remains shorter than the advanced world. Eradication of poverty and improvements in female employment and household income are the factors driven life expectancy higher over the last decade. To approach better health status in Malaysia, the results of this study suggest poverty completely eliminated, while unemployment rates should be reduced as much as economically possible. Moreover, higher household income would also enhance national health in Malaysia.

## Ethical considerations

Ethical issues (Including plagiarism, informed consent, misconduct, data fabrication and/or falsification, double publication and/or submission, redundancy, etc.) have been completely observed by the authors.
